# Self‐expanding stent effects on radiation dosimetry in esophageal cancer

**DOI:** 10.1120/jacmp.v14i4.4218

**Published:** 2013-07-08

**Authors:** Samual R. Francis, Christopher J. Anker, Brian Wang, Greg V. Williams, Kristen Cox, Douglas G. Adler, Dennis C. Shrieve, Bill J. Salter

**Affiliations:** ^1^ Department of Radiation Oncology University of Utah, Huntsman Cancer Hospital Salt Lake City Utah USA; ^2^ Division of Gastroenterology Department of Medicine, University of Utah Salt Lake City Utah USA

**Keywords:** esophageal cancer, stents, radiation, dosimetry

## Abstract

It is the purpose of this study to evaluate how self‐expanding stents (SESs) affect esophageal cancer radiation planning target volumes (PTVs) and dose delivered to surrounding organs at risk (OARs). Ten patients were evaluated, for whom a SES was placed before radiation. A computed tomography (CT) scan obtained before stent placement was fused to the post‐stent CT simulation scan. Three methods were used to represent pre‐stent PTVs: 1) image fusion (IF), 2) volume approximation (VA), and 3) diameter approximation (DA). PTVs and OARs were contoured per RTOG 1010 protocol using Eclipse Treatment Planning software. Post‐stent dosimetry for each patient was compared to approximated pre‐stent dosimetry. For each of the three pre‐stent approximations (IF, VA, and DA), the mean lung and liver doses and the estimated percentages of lung volumes receiving 5 Gy, 10 Gy, 20 Gy, and 30 Gy, and heart volumes receiving 40 Gy were significantly lower (p‐values <0.02) than those estimated in the post‐stent treatment plans. The lung V5, lung V10, and heart V40 constraints were achieved more often using our pre‐stent approximations. Esophageal SES placement increases the dose delivered to the lungs, heart, and liver. This may have clinical importance, especially when the dose‐volume constraints are near the recommended thresholds, as was the case for lung V5, lung V10, and heart V40. While stents have established benefits for treating patients with significant dysphagia, physicians considering stent placement and radiation therapy must realize the effects stents can have on the dosimetry.

PACS number: 87.55.dk

## I. INTRODUCTION

Esophageal cancer is a highly lethal and increasingly incident malignancy. It is estimated that 17,460 men and women were diagnosed and 15,070 people died from esophageal cancer in 2012.^(1)^ Standard of care treatment for locoregionally advanced disease (i.e., ≥T3 or node positive) involves concurrent chemoradiotherapy, which is either done in the neoadjuvant setting^(2)^ or as definitive therapy^(3)^ to improve overall survival. This disease often causes malignant dysphagia, which may require intervention with a feeding tube or esophageal self‐expanding stent (SES) if the obstruction prevents adequate nutrition. Due to the ease of use and demonstrated benefit of rapidly improving dysphagia, SESs are a primary and increasingly popular mode of treating malignant dysphagia.^4^, ^5^, ^6^, ^7^, ^8^ Although SESs are highly effective and safe, complications including chest pain, stent migration, perforation, and recurrent stent obstruction can occur.^4^, ^5^, ^6^, ^7^

Given the increasing use of esophageal SESs to treat dysphagia, many patients requiring radiation therapy have a stent in place prior to treatment. An important goal in radiotherapy is to minimize radiation‐induced toxicity by delivering sufficient dose locally to the tumor while minimizing radiation to organs at risk (OARs). When a combination of SES use and radiotherapy is employed, the inclusion of a stent in the radiation field introduces important questions regarding tumor and OAR dosimetry, as well as patient safety.

In 2002, Li et al.^(9)^ calculated the dosimetric perturbations caused by the presence of metallic stents in the treatment with single external photon beam and brachytherapy. Li and colleagues used two different stent simulation models and Monte Carlo techniques to make these calculations. They found that for single external photon beam treatment and brachytherapy, metallic stents caused local dose enhancement, resulting in a 5%–10% overdose to the esophageal mucosa in close proximity (0.5 mm) to the stent. Later, Atwood et al.^(10)^ created an experimental model, using a liquid water phantom, to measure dose perturbations caused by esophageal stents in the setting of single external photon beam radiotherapy. They examined three metallic stents and one nonmetallic stent. Atwood and colleagues found that the metallic stents produced the largest dose perturbations with distinct patterns of hot and cold spots. In another study, Chen et al.^(11)^ similarly measured the effects of esophageal stents on dose perturbations using a solid water phantom; however, in addition to a single AP (anteroposterior) photon beam, they measured dual parallel opposed beams (AP/PA). They found that by using multiple beams, the net impact of the stent on local dose enhancement could be reduced, thus making dose reduction unnecessary when providing radiotherapy in the setting of esophageal SESs.

While these studies examined the localized dose perturbations due to esophageal stents in the radiation field, they did not examine whether the physical presence of SESs affected the size of the planning target volume (PTV) or OAR dosimetry. We hypothesized that because stenting increases the lumenal diameter of the esophagus, stent placement would also increase the PTV, thus leading to an increased dose to OARs. The aim of this study was to quantify the impact of esophageal SESs on the PTV and the radiation dose delivered to OARs in patients with esophageal cancer.

## II. MATERIALS AND METHODS

Ten patients with esophageal cancer were evaluated, for whom a SES was placed before radiation. Patient inclusion required a computed tomography (CT) simulation scan with the SES in place and a CT scan done before the stent was placed. Esophageal cancer characteristics were obtained (Table 1). Tumor histology was adenocarcinoma in 90% of the patients, and 90% had involvement of the distal esophagus. The cancer stages^(12)^ ranged from IIB to IIIC, with 80% of patients being node positive.

**Table 1:** Patient characteristics

*Patient*	*Tumor Type*	*Tumor Location*	*Distance from Incisors (cm)*	*Node Location (PET and/or EUS)*	*T*	*N*	*M*	*Overall Stage*
A	Adenocarcinoma	Lower Thoracic into Cardia of Stomach	39–42	None	3	0	0	IIB
B	Adenocarcinoma	Lower Thoracic into Cardia of Stomach	35‐42	Three paraesophageal (EUS)	3	2	0	IIIB
C	Adenocarcinoma	Lower Thoracic	30–35	Six in paraesophageal, subcarinal, and perigastric areas (EUS); One right retrocrural and one right paratracheal (PET)	3	3	0	IIIC
D	Adenocarcinoma	Lower Thoracic/GE Junction	35–41	Three paraesophageal (EUS)	3	2	0	IIIB
E	Adenocarcinoma	Middle‐Lower Thoracic	27–32	Three paraesophageal (EUS); Two subcarinal (PET)	3	2	0	IIIB
F	Squamous Cell Carcinoma	Middle‐Lower Thoracic	25‐34	Two subcarinal (PET)	3	1	0	IIIA
G	Adenocarcinoma	Lower Thoracic	30–38	None	3	0	0	IIB
H	Adenocarcinoma	Lower Thoracic into Cardia of Stomach	34–38	Three paraesophageal (EUS)	3	2	0	IIIB
I	Adenocarcinoma	Lower Thoracic/GE Junction	38–45	Four paraesophageal (EUS)	3	2	0	IIIB
J	Adenocarcinoma	Middle Thoracic	25‐29	One paraesophageal (EUS/PET); Two left supraclavicular (PET)	3	2	0	IIIB

### A. ct imaging and fusion

All CT simulation scans were performed using our standard imaging protocols and GE LightSpeed RT 16‐slice large bore scanner (GE Healthcare, Waukesha, WI). Patients were positioned supine, with arms extended overhead and supported by Alpha Cradle (Smithers Medical Products Inc., North Canton, OH) or Vac‐Lok immobilization (Elekta‐Medical Intelligence, Atlanta, GA). Oral contrast was given before the scan. CT image voxel size was 1mm×1mm×2.5mm. PTVs and OARs (e.g., heart, lungs, liver, kidneys, and spinal cord) were contoured according to the Radiation Therapy Oncology Group (RTOG) 1010 protocol (version update December 20, 2011) using the Eclipse v10.0 (Varian Medical Systems, Palo Alto, CA) treatment planning system (TPS).

Diagnostic CT images obtained before SES placement (pre‐stent) involved patients positioned supine, eight patients with arms positioned at their sides, and two with arms positioned overhead. Pre‐ and post‐stent CT scans were manually fused by aligning the gross tumor volume (GTV), esophagus, heart, lungs, and bony anatomy using the Eclipse TPS image registration function. When anatomic variations between image sets existed, the GTV structure received alignment priority, with the other structures subsequently aligned to the best possible overall fit. On average, the pre‐stent GTVs overlapped the post‐stent GTVs by 87%±12% (standard deviation (SD)). We ensured that the superior aspect of each GTV was registered to the same axial plane for both CT image sets because the superior portion of the treatment volume has the greatest effect on lung and heart doses. PTVs and OARs were then contoured on the pre‐stent CT images. All CT fusions and contoured structures were approved by a radiation oncologist (CJA).

### B. creation of pre‐stent PtV approximations

Post‐stent dosimetry for each patient was compared to approximated pre‐stent dosimetry using three methods to represent pre‐stent PTVs: 1) image fusion (IF), 2) volume approximation (VA), and 3) diameter approximation (DA). Details for each method are noted in the following sections.

#### B.1 Image fusion (IF) method

For the IF approach, the previously contoured pre‐stent structures were superimposed on the post‐stent CT simulation scan after image fusion and then were used for radiation treatment planning.

#### B.2 Volume approximation (VA) method

**Figure 1:**
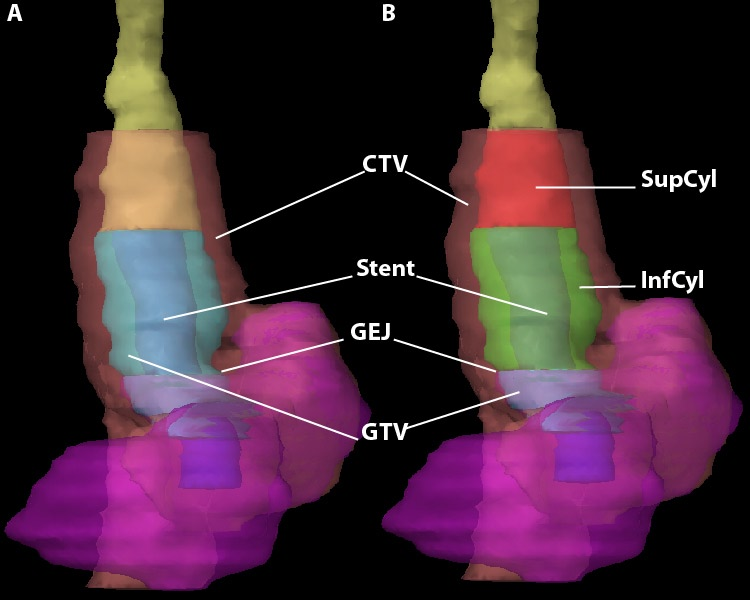
Coronal volume 3D reconstruction (a) showing the esophagus (yellow), with a self‐expanding stent (SES blue) extending past the gastroesophageal junction (GEJ) into the stomach (magenta) with the GTV (cyan) and CTV (pink) visible; Coronal volume 3D reconstruction (b) now showing post‐stent SupCyl and InfCyl structures (red and green, respectively). When fusing the pre‐stent to the post‐stent CT scans, priority was given to overlaying the SupCyl and InfCyl volumes of each scan, especially in the craniocaudal direction.

For both the VA and DA methods, two new volumes were defined: superior cylinder (SupCyl) and inferior cylinder (InfCyl) (Fig. 1). InfCyl is a composite of the GTV and esophagus volumes, with the distal border being the gastroesophageal junction (GEJ) and the proximal border being the superior end of the SES. SupCyl is part of the volume of the esophagus with the distal border being the superior slice of InfCyl and the proximal border being the superior slice of the original CTV. These volumes were created on the pre‐ and post‐stent CTs; however, the pre‐stent volumes were aligned to match the superior and inferior border of the post‐stent SupCyl and InfCyl.

**Figure 2:**
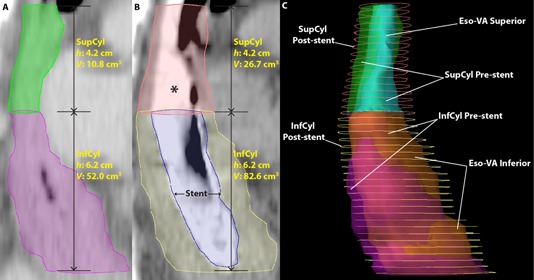
Volume approximation structures. CT sagittal image pre‐stent (a) showing SupCyl (green) and InfCyl (magenta), with height (h) and volume (V) shown. CT sagittal image of same patient (b) after self‐expanding stent (SES: blue) placement showing SupCyl (pink) and InfCyl (yellow), with height (h) and volume (V) shown. Both the SupCyl and InfCyl post‐stent volumes are larger than the pre‐stent volumes due to SES‐induced esophageal expansion. The * denotes contrast media in the esophagus. The proximal end of the stent (blue) ends at the top of InfCyl. Right lateral 3D reconstruction (c) showing the differences in volumes between the pre‐stent, post‐stent, and VA structures. The post‐stent volumes (contours, SupCyl: pink, InfCyl: yellow) are visibly larger than the pre‐stent volumes (transparent, SupCyl: green, InfCyl: magenta) and the VA structures (Eso‐VA Superior: cyan, Eso‐VA Inferior: orange) more closely approximate pre‐stent volumes.

For the VA method, volumes of SupCyl and InfCyl on the pre‐ and post‐stent CTs were calculated using Eclipse and then compared. The superior to inferior heights (cm) of these volumes were measured on the CT images (Fig. 2). Having the volume (V), height (h), and approximating the GTVs as cylinders ($V=\pi r^{2}h$), we calculated a radius (r), which allowed us to calculate a difference in radii between the pre‐ and post‐stent volumes. The post‐stent SupCyl and InfCyl dimensions were altered by this difference in radii to form new esophageal volumes, Eso‐VA Superior and Eso‐VA Inferior, and to ultimately make a new PTV (PTV‐VA).

#### B.3 Diameter approximation (DA) method

**Figure 3:**
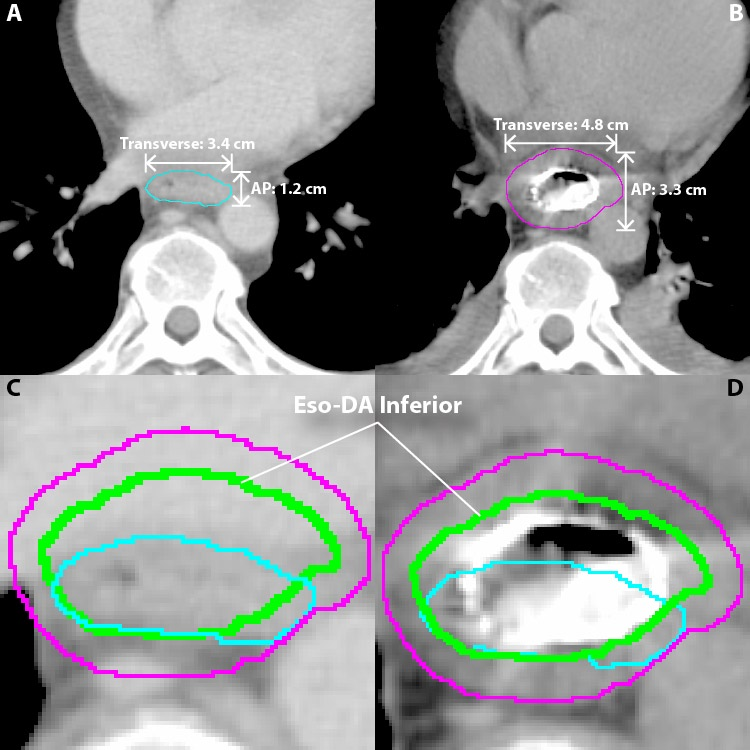
Diameter approximation (DA) structures. The DA esophageal volume (green) is based on the difference in the AP and transverse measurements of the pre‐ (cyan) and post‐ (magenta) InfCyl structures. For the DA method, width of the pre‐ and post‐stent InfCyl (shown) and SupCyl (not shown) volumes were measured in the anterior/posterior (AP) and transverse directions on the superior, middle, and inferior slices of each respective volume. An average diameter difference for each of the three values was calculated for both the AP and transverse dimensions. The post‐stent SupCyl and InfCyl AP and transverse diameters were then altered by their respective differences to create new esophageal volumes, Eso‐DA Superior (not shown) and Eso‐DA Inferior (shown), to ultimately make a new PTV (PTV‐DA) (not shown). Axial CT (a) showing the AP and transverse measurements of the pre‐stent SupCyl structure (cyan). Axial CT (b) showing the AP and transverse measurements of the post‐stent SupCyl structure (magenta). For this patient on this slice stent placement increased the AP dimension by 2.1 cm and the transverse dimension by 1.4 cm. Pre‐stent CT axial section (c) now showing the Eso‐DA Inferior structure (green). Post‐stent CT axial section (d) now showing the Eso‐DA Inferior structure (green). InfCyl pre‐stent (cyan); InfCyl post‐stent (magenta); Eso‐DA Inferior (blue); Eso‐VA Inferior (green).

**Figure 4:**
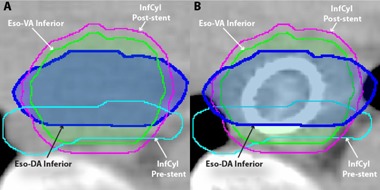
Comparison between VA and DA methods: (a) pre‐stent axial CT; (b) post‐stent axial CT. Note that for more oval‐shaped pre‐stent volumes such as this, the DA method may more closely approximate the pre‐stent esophageal contour.

For the DA method, diameters of the pre‐ and post‐stent SupCyl and InfCyl were both measured in the AP and lateral directions on the superior, middle, and inferior slices (Fig. 3). An average diameter difference between the pre‐ and post‐stent images was calculated for both dimensions. The post‐stent SupCyl and InfCyl were then altered by these differences to create new esophageal volumes, Eso‐DA Superior and Eso‐DA Inferior, and ultimately make a new PTV (PTV‐DA). The development of the DA method was fueled by the fact that the VA method, with its cylindrical approximation model, did not optimally approximate pre‐stent volumes when the esophageal lumen was oval‐shaped (Fig. 4).

### C. Dosimetry planning

**Figure 5:**
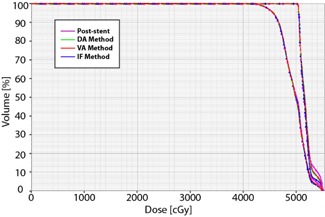
Dose‐volume histograms for PTV Initial and PTV Boost. As seen here, the dose‐volume histograms for the PTV Initial (left curve) and PTV Boost (right curve) are essentially the same for the post‐stent and all pre‐stent approximation methods. Also, it is noticeable that ≥95% of the PTV Initial received ≥95% of the prescription dose of 45 Gy, and that ≥99% of PTV Boost received ≥99% of boost dose of 50.4 Gy.

All dosimetry planning was completed by a certified medical dosimetrist (GW). Calculations were performed based on the CT simulation scans with the patients in the proper treatment position (e.g., arms up). Plans were optimized using the post‐stent structures and dose constraints based on RTOG 1010 (version update December 20, 2011) (Table 2). Per the protocol, the initial PTV received a dose of 45 Gy in 1.8 Gy fractions, and the boost PTV received 5.4 Gy for a total of 50.4 Gy. The celiac nodes were included in the initial PTV for patients with involvement of the lower thoracic esophagus. Plans were optimized so that 95% of the initial PTV received a minimum of 95% of the dose, and 99% of the boost PTV received at least 99% of dose (Fig. 5). Three‐dimensional conformal radiotherapy techniques were used for all plans. All patients were planned with four fields (AP, PA, and right and left laterals), except for patient 1 whose plan involved five fields (gantry angles: 0°, 70°, 140°, 180°, and 260°). A treatment energy of 10 MV was used for all beams for all patients, except for patient B, for whom the AP and PA fields’ energy were 18 MV, and the left and right lateral fields’ energy were 6 MV. The analytic anisotropic algorithm (AAA) in Eclipse was used for heterogeneity corrections. At our institution, we use weekly megavoltage portal images as image guidance for our esophageal patient treatment. As a routine daily QA program, therapists visually check the proper alignment of reticule.

**Table 2:** OAR dose constraints

*Organ*	*Volume Evaluated*	*Dose Constraint*
Total Lung	<60%	5 Gy
Total Lung	<40%	10 Gy
Total Lung	<25% (up to 30% allowed)	20 Gy
Total Lung	<20%	30 Gy
Total Lung	Mean	<20Gy
Heart	<50%	40 Gy
Heart	<100%	30 Gy
Liver	Mean	<25Gy
Liver	<40%	30 Gy
Spinal Cord	<0.01cc	45 Gy
Total Kidney	<30%	20 Gy

For the pre‐stent approximation methods, the previously designed field arrangement for the post‐stent plans were fit to the new PTVs (PTV‐IF, PTV‐VA, and PTV‐DA). Dosimetry data for OARs were recollected for the new pre‐stent plans. For the IF method, the heart dosimetry was gathered using pre‐stent contours to avoid inaccuracies in dosimetry. Specifically, we were concerned with changes in the size of the GTV and/or esophagus because of the stent altering the adjacent heart positioning due to differences in extrinsic compression. Additionally, separate heart structures were contoured for both the DA and VA methods in order to compensate for the displacement of the heart due to stent placement. To do this, the post‐stent heart volume was expanded posteriorly so that it abutted the GTV for the respective DA or VA method. Otherwise, the IF, DA, and VA methods evaluated OARs contoured from the post‐stent images. Lung volume was defined as a single, paired organ (total lung tissue) that excluded only the GTV. Dosimetry planning on post‐stent PTVs was used as a control for comparison.

### D. Stent measurements

The outside diameter of the stent was measured on the post‐stent CT images in the AP and lateral dimensions in the middle of the GTV in the craniocaudal dimension. To minimize oblique measurement error, measurements were performed by viewing the stent from axial, sagittal, and coronal planes.

### E. Statistics

Descriptive statistics (minimum, maximum, median, mean, and SD) and t‐tests (Microsoft Excel, Microsoft Office 2008) were used to compare post‐stent dosimetry data with dosimetry data obtained using the three described methods, as well as to compare stent size with changes in GTV diameter. A statistically significant difference was defined as p<0.05.

## III. RESULTS

Differences in target volumes are detailed in Table 3. Placement of a stent increased the GTV size by 30% on average, according to all the pre‐stent approximation methods. The PTV was affected more modestly by SES placement, increasing only 13%, 8%, and 8% using IF, VA, and DA pre‐stent approximations, respectively.

In addition to the mean dose received by the OARs, the dose‐volume percentages for OARs were assessed. For dose‐volume percentages, we used the nomenclature where “Vx” represents the tissue volume percentage receiving ≥x Gy. For example, when using the terms of V5, V10, V20, and V30, this refers to the percent of the OAR volume receiving doses of at least 5 Gy, 10 Gy, 20 Gy, and 30 Gy, respectively.

**Table 3:** Differences in target volumes between plans

	*Post‐stent Average Volume* $\pm \text{SD}({\text{cm}}^{3})$	*IF Average Volume* $\pm \text{SD}({\text{cm}}^{3})$	*% IF Difference From Post‐stent*	*VA Average Volume* $\pm \text{SD}({\text{cm}}^{3})$	*% VA Difference From Post‐stent*	*DA Average Volume* $\pm \text{SD}({\text{cm}}^{3})$	*% DA Difference From Post‐stent*
GTV	119±64	84±45	−29(p=0.006) ^a^	83±42	−30(p=0.004) ^a^	83±39	−30(p=0.007) ^a^
CTV	609±105	509±106	−16(p=0.001) ^a^	548±84	−10(p=0.002) ^a^	547±79	−10(p=0.004) ^a^
PTV	1105±151	965±163	−13(p=0.003) ^a^	1016±128	−8(p<0.001) ^a^	1016±119	−8(p<0.001) ^a^
PTV Boost	273±109	216±82	−21(p=0.005) ^a^	220±81	−19(p=0.002) ^a^	220±75	−19(p=0.004) ^a^

Note: A negative difference value denotes a decrease, while a positive denotes an increase, with respect to the post‐stent average values.

a =statistically significant; SD=standard deviation; IF=image fusion method; VA=volume approximation method; DA=diameter approximation method; GTV=gross tumor volume; CTV=clinical target volume; PTV=planning target volume.

**Figure 6:**
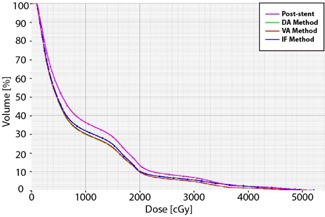
Lung dose‐volume histograms for a typical patient. The post‐stent lung dose‐volume percentages (magenta) are greater than those of each of the pre‐stent approximations (DA method (green), VA method (red), and IF method (blue)). This difference is most apparent at lower doses.

For each of the three pre‐stent approximation methods, the estimated mean lung dose and dose‐volume percentages were significantly lower than the corresponding post‐stent values (Table 4 and Fig. 6). This was true for each of the evaluated lung dose‐volume metrics. For IF, VA, and DA methods, the average V5 and V10 for lung were 6% and 8% lower, respectively, than the post‐stent V5 and V10. The lung V20 for IF, VA, and DA was 12%, 13%, and 13% lower than the post‐stent V20, respectively.

**Figure 7:**
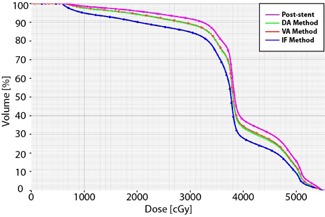
Heart dose‐volume histograms for a typical patient. The post‐stent heart dose‐volume percentages (magenta) are greater than those each of the pre‐stent approximations (DA method (green), VA method (red), and IF method (blue)).

**Figure 8:**
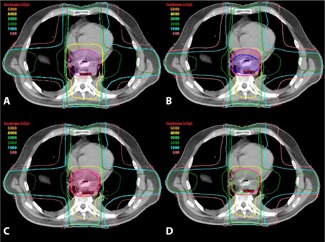
Axial isodose distributions for the post‐stent, IF, VA, and DA plans, showing axial dosimetric images on the same transverse section for a typical patient: (a) isodose distribution for the post‐stent plan with post‐stent PTV (magenta); (b) isodose distribution for the IF plan with the IF PTV (blue) and the post‐stent PTV (magenta) for comparison. Note that the IF PTV plan involves less volume of the heart receiving high doses. Isodose distribution (c) for the VA plan with the VA PTV (red) and the post‐stent PTV (magenta), and for the DA plan (d) with the DA PTV (lime green) and the post‐stent PTV (magenta). Notice the dose to the heart and lungs is higher in the post‐stent (a) than any of the pre‐stent approximations ((b) – (d)). The IF plan shows the lowest dose to the heart and lungs.

Using the DA and VA methods, the estimated pre‐stent heart V30 and V40 were significantly lower than the estimated V30 and V40 post‐stenting. Using the IF method, the estimated heart V40 was significantly lower than the post‐stent V40, while the V30 was not. The average heart V40 for IF, VA, and DA was 10%, 9%, and 9% lower than the post‐stent V40, respectively. A dose‐volume histogram for the heart is shown in Fig. 7. As shown in Fig. 8, higher isodose levels covered more of the heart and lungs for the post‐stent than the pre‐stent plans.

**Table 4:** Differences in OAR dosimetry compared to post‐stent plan

	*Metric (units)*	*Post‐stent Average Value* ±SD	*IF Average Value* ±SD	*% IF Difference From Post‐stent*	*VA Average Value* ±SD	*% VA Difference From Post‐stent*	*DA Average Value* ±SD	*% DA Difference From Post‐stent*
Lungs	V5 (%)	64±20	60±20	−6(p<.001) ^a^	60±21	−6(p<0.001) ^a^	60±21	−6(p<0.001) ^a^
	V10 (%)	49±18	45±18	−8(p<0.001) ^a^	45±18	−8(p<0.001) ^a^	45±18	−8(p<0.001) ^a^
	V20 (%)	20±8	18±8	−12(p=0.006) ^a^	17±8	−13(p<0.001) ^a^	17±8	−13(p=0.001) ^a^
	V30 (%)	13±8	12±7	−12(p=0.020) ^a^	11±7	−15(p<0.001) ^a^	11±7	−15(p=0.005) ^a^
	Mean (Gy)	13±4	12±4	−8(p=0.004) ^a^	12±4	−9(p<0.001) ^a^	12±4	−9(p=0.001) ^a^
Heart	V40 (%)	51±23	45±23	−10(p=0.004) ^a^	46±24	−9(p=0.002) ^a^	46±24	−9(p=0.003) ^a^
	V30 (%)	72±18	70±17	−3(p=0.147)	68±20	−6(p=0.003) ^a^	68±20	−5(p=0.018) ^a^
Liver	Mean (Gy)	15±6	14±6	−3(p=0.040) ^a^	15±6	−2(p=0.006) ^a^	15±6	−1(p=0.021) ^a^
	V30 (%)	13±7	13±8	+3(p=0.514)	13±7	−4(p=0.023) ^a^	13±7	−3(p=0.032) ^a^
Kidneys	V20 (%)	10±9	7±8	−18(p=0.054)	9±9	−1(p=0.196)	9±9	−2(p=0.191)

Note: A negative difference value denotes a decrease, while a positive denotes an increase, with respect to the post‐stent average values.

a =statistically significant; SD=standard deviation; IF=image fusion method; VA=volume approximation method; DA=diameter approximation method.

For both the DA and VA methods, the estimated pre‐stent liver V30 and liver mean dose were significantly less than the estimated post‐stent values, while only the mean liver dose was significantly lower with the IF method. All of the methods, including the post‐stent, had the same average maximum spinal cord dose of 43±3Gy. None of the methods showed a significantly lower pre‐stent dosimetry values for the kidneys compared to the post‐stent values.

The median stent diameter preplacement was 18 mm (Table 5). The median stent diameter based on CT image measurements at the middle of the GTV in the craniocaudal dimension was 17.6 mm in the AP dimension and 19.4 mm in the lateral dimension. These AP and lateral‐measured stent diameters were larger than the post‐stent esophageal diameter changes based on the VA method and DA methods.

**Table 5:** Stent sizes and change in GTV as measured on pre and post stent CT images

*Patient*	*Pre‐stent GTV (cm* ^*3*^ *)*	*Post‐stent GTV (cm* ^*3*^ *)*	*Stent Type*	*Fully or Partially Covered*	*Stent Size* (diameter×length) *(mm)*	*Measured Stent AP Diameter (mm)*	*Measured Stent Lateral Diameter (mm)*	*VA‐calculated GTV Diameter Change (both AP & Lateral) (mm)*	*DA‐Calculated GTV Diameter Change AP (mm)*	*DA‐Calculated GTV Diameter Change Lateral (mm)*
A	96.7	91.5	Alimaxx‐E	Fully	18×120	22.0	20.0	15.6	14.2	11.5
B	91.1	117.5	Alimaxx‐E	Fully	18×120	17.7	23.1	8.7	10.4	7.4
C	73.0	94.6	Alimaxx‐E	Fully	16×120	15.6	19.6	4.5	4.2	1.4
D	62.1	57.9	WallFlex	Fully	18×103	14.1	14.7	2.8	11.6	−9.5
E	34.3	75.0	Ultraflex	Partially	18/23proximal flare×100	17.5	19.1	8.8	8.7	9.3
F	150.0	248.0	Ultraflex	Fully	18×120	19	21.7	9.7	7.2	16.4
G	29.9	51.7	WallFlex	Fully	18×103	16.7	17.7	8.1	8.6	5.4
H	65.0	104.9	Polyflex	Partially	16×120	18.0	18.0	11.2	4.7	13.2
I	70.2	130.7	EndoMaxx	Fully	23 X 120	22.1	24.8	13.9	16.1	12.3
J	169.5	212.9	WallFlex	Fully	18×103	17.0	18.0	5.6	5.6	5.1
Median	71.6	99.8	n/a	n/a	18×120	17.6	19.4	8.8	8.7	8.4

Note: A positive change in diameter of the GTV denotes an increase in size post‐stent, while a negative denotes a decrease, with respect to the original GTV diameter. GTV=gross tumor volume; AP=anterior−posterior dimension; VA=volume approximation method; DA=diameter approximation method.

## IV. DISCUSSION

Our study is the first and only study to our knowledge that addresses how placement of esophageal SESs in patients with esophageal cancer affects PTV geometry and radiation dose delivered to surrounding organs (i.e., OARs). We found that esophageal SES placement leads to a significantly higher lung V5, V10, V20, V30, mean lung dose, heart V40, and mean liver dose compared to pre‐stent dosimetry. This was true for all pre‐stent approximation methods. Additionally, the potentially more accurate VA and DA pre‐stent approximation methods showed significantly higher heart V30 and liver V30 with stent placement. This study also found that meeting the lung V5 and heart V40 dose constraints was more easily attainable on average with pre‐stent target volumes (Tables 2, 4).

Though seemingly intuitive that stent placement, which dilates the esophagus, would increase the target dose field, this is the first study that addresses and quantifies the dosimetry differences between pre‐ and post‐stent placement. To ensure valid results, we developed three separate methodological approaches to examine our study aim, the impact of SESs on the PTV, and the radiation dose delivered to OARs. Each method was developed to approximate how the esophagus/tumor was altered due to SES placement. All methods relied on a comparison between pre‐ and post‐stent CT images.

The initial method used to address the study aim was the IF approach. This method found that the average pre‐stent lung dose metrics were all significantly lower than the post‐stent dose metrics (Table 4). As noted in the National Comprehensive Cancer Network (NCCN) Clinical Practice Guidelines for Esophageal Cancers (version 2.2012), consensus for optimal criteria for lung DVH parameters as predictors of pulmonary complications has not been established and is still an active area of research. Although the NCCN guidelines recommend minimizing all lung dosimetric parameters, the doses constraints outlined in RTOG 1010 (Table 2) highlight some of those that are believed to be most important according to the current literature. The lung V20 was perhaps the first dose constraint to emerge as highly associated with development of pneumonitis in patients receiving concurrent chemoradiation,^13^, ^14^ leading to the recommendation of V20<25%, as used in RTOG 1010. While almost all of the post‐stent patient plans met this constraint, all of the pre‐stent plans had a significantly lower V20. Lee et al.^(15)^ found increased postoperative pulmonary complications (e.g., pneumonia and acute respiratory distress syndrome) if the preoperative pulmonary V10 was ≥40%, and therefore this recommendation is used as an RTOG 1010 dose constraint. As the majority of plans in our study exceeded this limit on average, the 8% decrease in the V10 for the pre‐stent as compared to the post‐stent plan is all the more important to help decrease the risk of toxicity.

Wang et al.^(16)^ also looked at the postoperative pulmonary complications in esophageal cancer patients treated with concurrent neoadjuvant chemoradiation. While they did find an association with mean lung dose and V5 with postoperative complications, the only independent predictor they reported for increased toxicity was the volume of lung spared from doses ≥5Gy(VS5). Therefore perhaps one of the most important ways to reduce risk of lung injury is to minimize the lung V5 as much as possible. The RTOG 1010 recommends V5<60% (Table 2). In our study, the post‐stent average V5 was 64%±20% and the pre‐stent IF average V5 approximation was 60%±20%. Thus, SES placement may increase the difficulty in meeting this dose constraint. In a subsequent study, Wang et al.^(17)^ found that systemic chemotherapy prior to definitive chemoradiation was the only significant risk factor for radiation pneumonitis, rather than any clinical or dosimetric factors. Similarly, the QUANTEC report for the lung toxicities,^(18)^ after extensive literature review of dose‐volume parameters and pneumonitis, found inconsistent results for predictive metrics. Based on this literature analysis, the report's authors found that a variety of dose‐volume thresholds (i.e., Vx values) are associated with radiation pneumonitis risk, and thus they could not establish a sharp dose‐volume threshold below which there was no risk of pneumonitis. They did report a positive correlation between increasing mean lung dose and probability of radiation pneumonitis, but did not observe a threshold or absolute “safe” mean lung dose below which there was no risk of radiation pneumonitis. Therefore, although the mean lung dose constraint was met, the 8% decrease for the IF method compared to post‐stent dosimetry could decrease pulmonary toxicity.

In the treatment of esophageal cancer, attempts to minimize lung dose is often met with increases in the cardiac dose. Although Wei et al.^(19)^ found a significantly increased risk of pericardial effusion for esophageal cancer patients with a V30>46%, RTOG 1010 recommends a V30<100%, as increased cardiac mortality has been found with whole heart doses above 30 Gy.^(20)^ Another RTOG 1010 dose constraint that was affected by stent placement was the heart V40<50%. Hancock et al.^(20)^ found an even higher risk of cardiac death with doses above 40 compared to 30 Gy,^(21)^ and the risk of chronic constrictive pericarditis has been found to increase significantly above 41 Gy.^(22)^ On average the heart V40 was 10% higher after stent placement using the IF approximation (Table 4), raising the average V40 from 45±23 to 51±23. This 10% increase in heart dose caused by the stent placement may make it more challenging to achieve the V40 dose constraint, thus increasing the risk of death from cardiac causes.

The IF method has limitations. Because positional and anatomical variations exist between the pre‐ and post‐stent CT images, it is impossible to fuse the images perfectly. As such, this method contained a margin of error that interfered with our ability to isolate and analyze the changes on target volumes and dose to OARs due specifically to stent placement.

In order to overcome the limitations of positional and anatomical variability of the IF method, the VA and DA methods were introduced. Both of these methods used measurements of the esophagus within the target volume on the pre‐ and post‐stent images to calculate dimensional modifications to the esophagus on the post‐stent structures. By directly measuring the anatomy of the same patient on the pre‐ and post‐stent images, then using this data to dictate modifications of the esophageal target volume, these methods overcame limitations of the IF method.

Consequently, they provided a more accurate approach to analyzing the effects of SESs on the target volumes and radiation dose to OARs. Both the VA and DA methods yielded very similar results. The pre‐stent VA and DA average lung doses were 6% (V5), 8% (V10), 13% (V20), 15% (V30), and 9% (mean) lower than the post‐stent doses (Table 4). As was seen with the IF method, the VA and DA average pre‐stent lung V5(60%±21%) was significantly lower than the post‐stent value (64%±20%). This gives further evidence that the placement of an SES increases the lung V5, making it potentially more difficult to meet this critical dose constraint. While the mean lung dose goal was met in all of the plans (Tables 2, 4), the pre‐stent mean lung doses were between 8%–9% lower than the post‐stent plans. As mentioned previously, since there is no clear toxicity threshold, any reduction in mean lung dose may be beneficial in preserving lung function. Also, as was seen with the IF method, the VA and DA average pre‐stent heart V40(46%±24%) was within tolerance for the recommended dose constraint while the average post‐stent heart V40(51%±23%) was not.

A potential limitation of the VA and DA methods is that by contracting the GTV and esophageal volumes to estimate the pre‐stent parameters without accounting for the heart's positional change due to the stent, one might underestimate the true heart dose because the stent displaces the heart anteriorly. To overcome this, we created a unique heart structure for each of the DA and VA methods. We expanded the post‐stent heart volume posteriorly to abut the corresponding VA or DA esophageal volume. This ensured that we did not underestimate the heart dose, and indeed provided a worst‐case heart dose approximation because we did not adjust the anterior border of the heart contour.

Based on this work, we believe that the lung V5 and heart V40 are the parameters for which placement of a stent may have the largest impact on meeting dose constraints. Though there were other dose constraints that had significant differences pre‐ and post‐stent placement (e.g. liver mean, lung V10, lung V20), only the lung V5 and heart V40 exceeded dose constraints on average after stent placement but met corresponding dose constraints using our pre‐stent approximations (Table 4). However, as mentioned previously, the clinical value in terms of toxicity and outcomes in meeting these dose constraints is still being more fully defined. As more data become available, SES impact on these dose constraints will allow clinicians to make better treatment decisions. In order to minimize OAR dose, one may hypothesize that patients might be better off from a radiation dosimetric point‐of‐view if they were to receive the smallest appropriate stent size that may still relieve their obstruction. The downsides to this argument from a clinical point‐of‐view include the fact that larger diameter stents are less likely to clog and/or migrate, may produce superior improvement in dysphagia, and have longer patency rates when compared to narrower stents. Overall, stent size selection should be individualized and is dependent on a variety of factors. Additionally, the effects of SESs on the lung V10 may have clinical importance. Although the lung V10 was often not met regardless of whether a stent was in place or not, each of the pre‐stent approximations estimated an 8% decrease in the V10 compared to post‐stent dosimetry.

Another finding in our study is that the addition of a stent never increased the outer esophageal diameter by the full diameter of the stent (Table 5). In other words, an 18 mm stent diameter only increased the esophageal/tumor diameter by a fraction of the stent size, and never by the full 18 mm. This is probably due to a combination of esophageal wall/tumor compression by the stent, and the fact that the esophageal lumen was incompletely occluded and could partially accommodate the stent. This is also likely due to the fact that most stents do not completely expand and have some degree of central narrowing at the point of greatest tumor involvement.

Additionally, we found that the stent has the largest relative effect on the GTV size and the least on the PTV (Table 3). Across all methods, the GTV was 30% larger after the placement of an SES, while the PTV increased much less. This is because the stent affects primarily the esophageal diameter, which only contributes to a portion of the overall CTV and PTV. Other areas included in the target volume, such as the stomach and celiac region, dilute the effects of the stent on the PTV expansion.

Our data clearly show that placement of SESs have a significant impact on certain dose constraints. One potential limitation about our conclusions is that the RTOG 1010 dose constraints are given as best practice guidelines, while the exact likelihood of complications, and thus the clinical impact, for these recommended dose constraints is still under investigation. Another limitation of our study is that ideally the pre‐ and post‐ stent CT scans would have been done with the patients’ arms in the same position in order to minimize anatomical differences between images. However, given that we used three different methods, two of which accounted for anatomical variations between images (VA and DA) and one that did not (IF), and still obtained very similar results, the validity of our methods and results is substantiated. Our study is also limited by being purely a dosimetric study with retrospectively designed dosimetry plans, and thus direct clinical correlations of toxicities and outcomes of our patients are not possible. In future work, we will consider the clinical outcomes and complications of esophageal cancer patients who receive radiotherapy and SESs.

## V. CONCLUSIONS

Our study shows that placement of esophageal SESs in patients with esophageal cancer increases the PTV and the radiation dose‐volume percentages for the lungs, heart, and liver. While these increases were only by a small amount overall, they may have clinical importance as dose constraint thresholds as predictors of organ toxicity are better elucidated. However, based upon current guidelines, our study suggests the parameters most likely to exceed current recommended thresholds due to stent placement are the lung V5 and heart V40. The effect of stents on dosimetry must be realized by physicians considering stent placement before radiation and those considering stent removal during radiation therapy. Given the effectiveness of stents to relieve symptoms of dysphagia in neoadjuvant and palliative care settings, we recommend consideration of stents when significant dysphagia is present, but clinicians should be aware of the effects these stents may have on dosimetry. However, the magnitude of the variation in dose‐volume metrics for the heart and lungs is unlikely to change current clinical practice.
